# Low-noise fluorescent detection of cardiac troponin I in human serum based on surface acoustic wave separation

**DOI:** 10.1038/s41378-023-00600-5

**Published:** 2023-11-09

**Authors:** Xuan Chen, Chuanyu Zhang, Xianglian Liu, Yangchao Dong, Hao Meng, Xianming Qin, Zhuangde Jiang, Xueyong Wei

**Affiliations:** 1 State Key Laboratory for Manufacturing Systems Engineering, Xi’an Jiaotong University, Xi’an, 710049 China; 2 Department of Microbiology, School of Preclinical Medicine, Fourth Military Medical University, Xi’an, 710032 China; 3 The Second Affiliated Hospital of Xi’an Jiaotong University, Xi’an, 710004 China; 4 School of Mechano-Electronic Engineering, Xidian University, Xi’an, 710071 China

**Keywords:** Chemistry, Engineering, Physics

## Abstract

Acute myocardial infarction (AMI) is a life-threatening disease when sudden blockage of coronary artery occurs. As the most specific biomarker, cardiac troponin I (cTnI) is usually checked separately to diagnose or eliminate AMI, and achieving the accurate detection of cTnI is of great significance to patients’ life and health. Compared with other methods, fluorescent detection has the advantages of simple operation, high sensitivity and wide applicability. However, due to the strong fluorescence interference of biological molecules in body fluids, it is often difficult to obtain high sensitivity. In order to solve this problem, in this study, surface acoustic wave separation is designed to purify the target to achieve more sensitive detection performance of fluorescent detection. Specifically, the interference of background noise is almost completely removed on a microfluidic chip by isolating microbeads through acoustic radiation force, on which the biomarkers are captured by the immobilized detection probe. And then, the concentration of cTnI in human serum is detected by the fluorescence intensity change of the isolated functionalized beads. By this way, the detection limit of our biosensor calculated by 3σ/K method is 44 pg/mL and 0.34 ng/mL in PBS buffer and human serum respectively. Finally, the reliability of this method has been validated by comparison with clinical tests from the nephelometric analyzer in hospital.

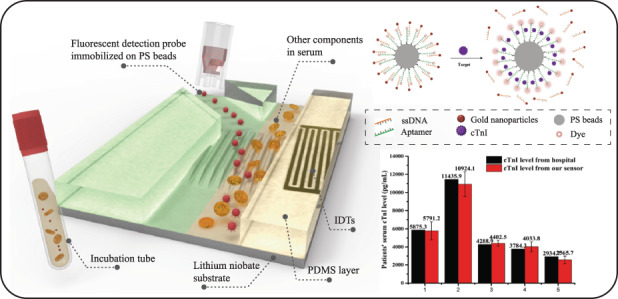

## Introduction

Acute myocardial infarction (AMI) is a serious form of cardiovascular diseases with high mortality rate^1^. According to WHO, AMI is considered as the leading cause of death globally^2^. AMI occurs due to myocyte necrosis resulting from acute obstruction of a coronary artery. Subsequently, cell membrane permeability is increased, leading to the release of biomarker proteins into bloodstream^3^. Among all the biomarker proteins, the level of cardiac thoroponin I (cTnI) is proved to be highly associated with the severity of heart muscle damage, and commonly regarded as the most sensitive and specific biomarker for AMI^4,5^. cTnI level will be detectable in AMI patients’ blood 4–6 h after myocyte necrosis, and the peak will show 14–48 h later, maintaining high level for up to 12 day^6^. The level of cTnI not only is an important diagnostic criterion for AMI, but also has great prognostic value of monitoring postoperative condition and predicting in-hospital mortality^7,8^. It is critical for patients to diagnose AMI as early as possible so that doctors can take further treatment steps^9^. Therefore, the accurate and rapid detection of cTnI level is crucial for AMI patients.

Many efforts have been made on this challenge, including electrochemical method^10,11^, surface-enhanced Raman spectroscopy (SERS) method^12,13^, field effect transistor (FET) method^14,15^, lateral flow immunoassay^16^ and fluorescent method^17–19^. Among all these methods, fluorescent biosensors have many advantages such as simple operation and readout instrument, high sensitivity and broad applicability. As an ultrasensitive method and gold standard, enzyme-linked immunosorbent assay (ELISA) has been adopted to quantify low level of cTnI. An improved ELISA immunoassay was proposed by Miao et al. based on fluorescence resonance energy transfer (FRET) and allochroic effect^17^. Liu’s group developed a polymer carbon dots based fluorescent ELISA method by detecting the activity of alkaline phosphatase (ALP)^18^. Recently, as a very competitive alternative to antibody in biosensing, aptamer has also been widely used due to many advantages including easier synthesis process, more stable production quality between batches, longer shelf time and excellent affinity and specificity to targets. For instance, a fluorescent aptasensor based on graphene oxide platform was proposed by Liu et al.^19^, where FAM-modified aptamer was adsorbed on the surface of graphene oxide through π–π stacking interaction. The excellent fluorescence quenching property of graphene oxide was also used by Li’s detection method based on rolling circle amplification^20^. Meanwhile, Rezaei et al. reported a self-assembled aptasensor based on FRET effect between CdSeS/ZnS quantum dots and gold nanoparticles^21^. Metal-enhanced fluorescence was utilized to quantify the concentration of cTnI in human saliva due to the conformational change of the linker DNA. Although fluorescent detection methods have been widely developed, as the complexity in samples, the bottleneck for the detection of cTnI is the strong fluorescence interference from background biological fluids, especially serum. In such, depressing noisy signals turns out to be a vital step to access accurate measurement.

Microfluidic technique provides the novel perspectives to break the limits for biosensors and has attracted attention from many researchers due to its capability of microscale manipulation, portability and good compatibility to incorporate with other techniques. A microfluidic device was proposed by Qiu et al. based on a Lys-AuNPs@MoS2 nanocomposite fluorescent immunoassay for cTnI detection^22^. Due to its superiority in gentle, label-free and selective manipulating particles based on mechanical parameters, like size, microfluidics based on surface acoustic wave (SAW), in recent years, have been used a lot for biological detection including protein^23^, cellular damage^24^, viruses^25^ and bacteria^26^, and multifunctional manipulation of microparticles including mixing^27^, focusing^28^, and separation^29^. Especially, SAW has proven its feasibility in separation of biomolecules including cells^30^, microorganism^31^, viruses^32,33^, bacteria^34^, and even exosomes^35^ from complex sample environment. For instance, our group employed aptamer-modified microspheres for the isolation of multi viruses on chip^33^. Devendran et al. successfully achieved focused E. coli bacteria cells in microfluidic channel based on diffractive-acoustic surface acoustic waves^34^. The ability of sorting biomolecules makes it possible to remove or decrease the source of background fluorescence noise by isolating the targeted cTnI by SAW. However, limited by the very weak acoustic response of nanoscale particle, direct isolation of proteins is quite challenging and more researchers, instead, tend to functionalize the low-compressibility micro-scale beads with selective binding probe on the surface to capture the proteins, making the separation process much easier. For example, Liu et al. utilized 40 μm SiO_2_ microbeads to amplify the size of circulating tumor cells for a high-purity cell separation from blood samples^36^. Yoon’s group has reported to successfully separate thrombin^37^ and three different proteins^38^ from blood based on aptamer modified polystyrene (PS) beads. This method allows to trap rare targets and easily complete the isolation by virtue of SAW. Moreover, the utilization of highly flexible and affordable polymer substrates such as polyimide (PI), polyethylene terephthalate (PET) and polyethylene naphtholate (PEN) paves the way for application of SAW technology in wearable devices^39,40^. For example, Leonardo et al. developed a flexible AlN/PEN SAW biosensors for the detection of E.Coli^41^, while Tao et al. integrated electromagnetic metamaterials with PI coated carbon fiber, demonstrating the capabilities of the SAW device for wireless,in situ and wearable biosensing^42^.

Herein, we propose a fluorescent aptasensor for the detection of cTnI based on travelling surface acoustic wave (TSAW) particle separation. PS beads immobilized with fluorescent aptamer probe will specifically capture the target in serum and the introduction of microfluidic chip using SAW technique can effectively separate the PS beads from complex body fluid, which will eliminate the background signal to a great extent. Due to the completely automated process of SAW particle separation, our system has enormous potential for sample-in-result-out device.

## Experimental methods

### Design of the acoustofluidic chip

SAW is a kind of elastic wave travelling along the surface of solid. The generation of SAW relies on a pair of interdigital transducers (IDTs) and a piezoelectric substrate. When an AC signal of a certain frequency is applied to both ends of IDTs, the piezoelectric substrate beneath the IDTs will vibrate due to the inverse piezoelectric effect, generating TSAW perpendicular to the direction of the IDTs. Since SAW only penetrates one wavelength in depth from the solid surface, its energy density is extremely high. The secondary nonlinear effect during the interaction between acoustic wave and fluid allows to manipulate small particles.

A time averaged acoustic radiation force (ARF) and acoustic streaming-induced drag force (ASF) will act on particles in an acoustic field simultaneously^43^. When SAW propagates through the medium containing suspensions inside, the scattering of wave from the surface of particles will induce ARF. The magnitude and direction of ARF are closely associated with the acoustic field itself and the mechanical properties of medium and particles. Ideally, for inviscid fluid and ${r}_{p}\ll \lambda$, the ARF acting on spherical particles can be calculated by the following formula^44^:

1 $${F}_{{rad}}=-\frac{4\pi }{3}{r}_{{p}}^{3}\left(\frac{{f}_{1}}{2{c}_{{f}}^{2}{p}_{{f}}}\bar{{p}^{2}}-\frac{3{f}_{2}}{4}{p}_{{f}}\bar{{u}_{{in}}^{2}}\right)$$

2 $${f}_{1}=1-\frac{{k}_{\rm{s}}}{{k}_{\rm{f}}}$$

3 $${f}_{2}=2\frac{{\rho }_{{p}}-{\rho }_{{f}}}{{2\rho }_{{p}}+{\rho }_{{f}}}$$

Where ${r}_{\rm{p}}$ and ${\rho }_{\rm{p}}$ are the radius and density of particles respectively. $\bar{{p}^{2}}$ and $\bar{{u}_{{{in}}}^{2}}$ represent the time average value of the square of the SAW pressure and the square of the fluid injection velocity in a single wave period. ${k}_{{{s}}}$ and ${k}_{{{f}}}$ represent compressibility coefficient of particles and fluid respectively. The formula describes the acoustic radiation force of a single particle in the fluid, ignoring the refraction and reflection from other particles in the fluid and the wall of microchannel.

In addition to ARF, particles in viscous fluid and far away from the microchannel wall will also experience Stokes drag force. When a particle with a radius of ${r}_{{{p}}}$ moves with a velocity of ${u}_{{{p}}}$ in fluid, the expression of Stokes drag force is:

4 $${F}_{{{drag}}}=-6\pi \mu {r}_{{{p}}}({u}_{{{p}}}-{u}_{{{f}}})$$

Where ${u}_{\rm{f}}$ is the velocity of fluid surrounding the particle and $\mu$ is the dynamic visocity of the fluids. The expression of Stokes drag force describes the drag force acting on particles in a laminar flow, when the size of particles is far smaller than the width of the channel. The ARF and Stokes drag force usually act on the particles and affect the motion of particles trajectory simultaneously.

A dimensionless factor ${\rm{\kappa }}={\rm{\pi }}{\rm{df}}/{c}_{\rm{f}}$ has been proposed to analyze the mechanism of particle manipulation in fluid based on TSAW^45^, where d is the diameter of particles and f is the frequency of acoustic wave. Previous research work has demonstrated that ASF will be the dominant force when *κ* < 1 because of the spherically isotropic scattering of particles, while ARF will be the dominant force when *κ* > 1 since the backscattering dominates and a net momentum transfer to the particle occurs^46^. For a certain microparticle, larger ${\rm{\kappa }}$ value means SAW device with higher resonant frequency should be adopted. However, with the increase of the resonant frequency of IDTs, the thermal effect would also become unignorable, which is adverse to the stability of the device. In our work, after many trials, we chose 50 MHz as the resonant frequency of the IDTs to balance the capabilities of manipulating particles and the thermal effect of the device. When the diameter of target particles is 10 μm, the factor ${\rm{\kappa }}$ can be calculated to be 1.05. By observation from experiments, with the current value of ${\rm{\kappa }}$, the disturbance of streaming flow is not observed during purification process by ARF. In addition, once the frequency of IDTs (mainly decided by the distance of neighboring metallic electrodes) is designed, deviation of resonant modes (whether increasing or decreasing the frequency) will weaken the effect of ARF on the particles, and the only factor we can change to control ARF is voltage for a certain target.

**Fig. 1:**
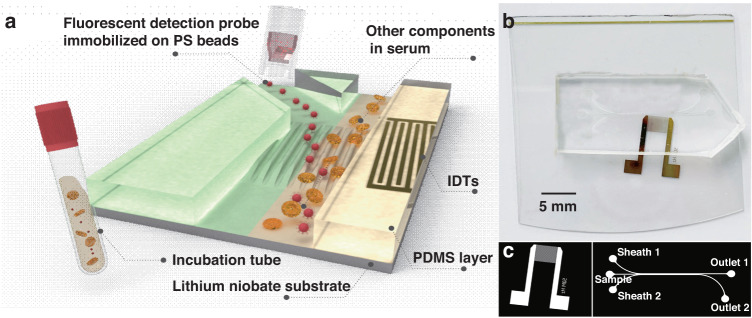
Schematic of proposed biosensor and chip design. **a** The schematic of SAW microfluidic chip. **b** SAW device fabricated by us. **c** 50 MHz IDTs designed. **d** Microfluidic chip designed

Since ARF is proportional to the cube of particle radius radius and is sensitive to the scattering efficiency of the particles, ARF can be used for size-dependent separation of microparticles. Compared with biological particles, PS beads correspond to lower compressibility and much larger scattering efficiency encountering incident acoustic wave. Thus, the larger-size PS beads can be effectively separated from mixed samples. The introduction of SAW based microfluidic chip in our work aims to isolate cTnI attached on the surface of PS beads and to eliminate the interference fluorescence from serum since the components in serum have strong fluorescence in a wide spectrum range. The schematic of our SAW microfluidic chip is shown in Fig. 1a.

The SAW device fabricated by us is shown in Fig. 1b, which consists of a lithium niobite substrate with IDTs fabricated on and a polydimethylsiloxane (PDMS) microchannel. The designed IDTs and PDMS microfluidic chip are shown in Fig. 1c, d respectively. The IDTs used by us have 30 pairs of electrodes. Both the width and pitch of our IDTs are designed to be 20 μm. The wavelength of TSAW generated by IDTs is then close to 80 μm. The microchannel is designed with three inlets. Inlets on both sides are used for sheath flows, while the inlet in the middle is used for injection with sample solution. By controlling the pressure of sheath flows, the width and position of the sample flow in the microchannel can be adjusted. The width of the main channel is 300 μm and a 200 μm width branch channel is designed to collect target particles separated by SAW. The width of the other branch channel keeps 150 μm.

The fabrication of the SAW device includes patterning IDTs on the lithium niobite substrate, fabricating the PDMS microchannel chip by soft-lithography technology and finally bonding the lithium niobite substrate with the PDMS microchannel chip. A double-side polished lithium niobate wafer with 128° Y-cut X-propagation (Shanghai Daheng Optics & FINE MECHANICS Co., Ltd, China) was selected as the piezoelectric substrate by us. To pattern IDTs on the lithium niobite substrate, the photoresist (EPG535, Everlight Chemical, Taiwan, China) was firstly spread on the substrate by spin coating. Then the substrate was placed under the mask with the pattern of IDTs for photolithography with Manual Mask Aligner System (ABM/6/350/NUV/DCCD/M, ABM Company, USA). The photoresist was removed by NaOH (0.5%, Acmec Biochemical, China). Then, 50 nm of Cr layer and 200 nm of Au layer were deposited by electron beam evaporation by a versatile box coater (TF500, HHV Ltd, UK). Due to the width and spacing distance of our IDTs are quite narrow (both are only 20 μm), the fabrication of intact IDTs is relatively difficult. To increase fabrication efficiency, the IDTs were designed oppositely on the lithium niobite substrate. Only one pair of IDTs was used in actual separation experiments.

The PDMS microchannel chip was fabricated by a standard soft-lithography technique. The microchannel mold was firstly fabricated by conventional photolithography. The SU-8 negative photoresist (2050, MicroChem Corp., USA) was then coated on the silicon substrate by spinner. After photolithography, the photoresist was removed by developer for negative photoresist. Subsequently, the PDMS prepolymer base (Sylgard 184, Dow Corning Corporation, USA) was mixed with the curing agent (w/w = 10:1). Bubbles in the mixture should be evacuated before pouring on the microchannel mold. After curing 2 h at 85 °C, the PDMS microchannel chip was peeled off carefully. Lastly, the PDMS microchannel chip and the lithium niobate were cut into proper size and treated with oxygen plasma for 30 s in a plasma cleaner (ZEPTO, Diener Electronic, Germany) for bonding. Since the microchannel chip and the lithium niobate were bonded manually under a microscope in our lab, the distance and angel between the microchannel and the IDTs varied slightly with each device. The angle between the microchannel and the IDTs is designed to be 7°. However, due to the manual bonding error, the actual angle is usually around 5° to 10°. Also, with keeping the IDTs outside the microchannel, the distance between the microchannel and the IDTs is designed to be as close as possible. The small distance would decrease the attenuation of SAW and the tilted IDTs help to isolate particles more efficiently.

To excite SAW and observe the separation process, the experimental platform is consisted of an electric signal generator, a power amplifier, a four-channel microfluidic control system, a microscope, a high-speed camera, a computer and a microfluidic chip. The electric signal generator (Agilent, 33250A, USA) and the power amplifier (BA4850, NF Corporation, Japan) were connected in series to generate SAW. The four-channel microfluidic control system (MFCS-EZ, Fluident, France), which was a combined high-precision microfluidic pressure pump could accurately control the pressure of the liquid injected into the microfluidic channel. The microscope (M330-M100, AOSVI, China), the high-speed camera (Phantom, MIROEX4-4096MC, USA) and the computer were used to observe the motion of particles in the microfluidic channel.

### Mechanism and synthesis of detection assay

**Fig. 2:**
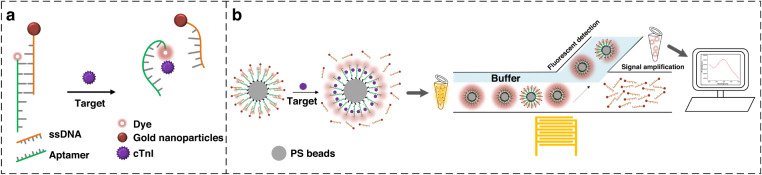
Detection mechanism and process. **a** The detection mechanism of competitive assay. **b** Detection process of our detection method

The detection mechanism of our competitive aptasensor is shown in Fig. 2a. A competitive assay consisting of a cTnI aptamer modified with fluorescent dye (Texas Red) and a ssDNA with a gold nanoparticle is adopted in our work. Since the ssDNA is complementary to a short segment of cTnI aptamer, the distance between the fluorescent dye modified on aptamer and the gold nanoparticles modified on ssDNA is extremely short. When the target shows up, cTnI aptamer will specifically bind with the target and release ssDNA due to a higher binding affinity. The detection assay is immobilized on the surface of 10 μm PS beads so that SAW device is able to separate them from the mixture (Fig. 2b).

Fluorescence resonance energy transfer (FRET) effect happens to a pair of extremely close molecules (usually less than 15 nm) under the condition where the emission spectrum of the donor and the absorption spectrum of the receptor overlap significantly. An electron transfer between the FRET pair will occur with an appropriate distance between donor and acceptor as well as a spectral overlap between donor emission and acceptor absorption. The efficiency of FRET effect can be evaluated by^47^:

5 $${E}_{{FRET}}=\frac{{k}_{{{D}}\to {{A}}}}{{k}_{{{D}}\to {{A}}}+{\tau }_{{D}}^{-1}}=\frac{{R}_{0}^{6}}{{R}_{0}^{6}+{R}^{6}}$$

Where *R*_*0*_ is the Förster distance, defined as the donor-acceptor distance with a FRET efficiency of 50%, and *R* is the actual donor-acceptor distance. The equation shows the efficiency of FRET effect is proportional with *R*^−6^. For the detection probe in this study, the fluorescence of dye is firstly quenched by gold nanoparticles due to the hybridization between cTnI aptamer and ssDNA. When cTnI appears, the increase of *R* induced by the releasing of ssDNA will attenuate the efficiency of FRET effect and the fluorescence of dye will be recovered. Thus, the concentration of cTnI in the sample can be characterized by the variations of fluorescence intensity compared with that of the standard sample.

The cTnI aptamer and ssDNA used in this work were synthesized and purified by HPLC from Shanghai Sangon Biotechnology (China). The aptamer sequence is biotin-5′ CGT GCA GTA CGC CAA CCT TTC TCA TGC GCT GCC CCT CTT A 3′-Texas Red. The ssDNA sequence is SH-C6-5′ GAG AAA GGT TGG CGT ACT 3′. The spacer DNA sequence is 5′ TTT TTT TTT T 3′-C6-SH. (2-carboxyethyl)phosphine (TCEP), N-hydroxysuccinimide (NHS), MES buffer (0.05 M, pH = 5.5) and phosphate-buffered saline (PBS, pH = 7.2), bovine serum albumin (BSA) and nuclease-free water were all purchased from Sangon Biotechnology. 1-Ethyl-3-(3-dimethylaminopropyl)carbodiimide (EDC) was bought from Thermo Scientific (USA). Gold(III) chloride hydrate, tween-20, chloroauric acid, tannic acid and sodium citrate were bought from Shanghai Acmec Biochemical (China). Human cardiac troponin I (cTnI) was bought from Guangzhou Wondfo Biotech (China). Streptavidin coated polystyrene (PS) beads was bought from Taizhou Bionano New Material Science Co.,Ltd. C-reactive protein (CRP) and myoglobin (MYO) were purchased from Cloud-clone Company (Wuhan). The cTnI antibody used was purchased from Proteintech (USA). Quartz cuvettes were bought from Aoruituo Optical Instruments Company (Yixing, China). All chemicals were used directly without further purification.

The transmission electron microscopy (TEM) observations were done on a JEM-2100 Electron Microscope (JEOL Ltd., Japan). UV–Vis absorption spectra was acquired on a Nicolet iS10 spectrometer (Thermo Fisher, U.S.). Fluorescence measurements were performed in a quartz cuvette with a path length of 1 cm at room temperature, through a FS5 Spectrofluorometer from Edinburgh Instruments. The absorption spectrum was obtained from Synergy™ 2 Multi-Mode Microplate Reader from Biotek.

**Fig. 3:**
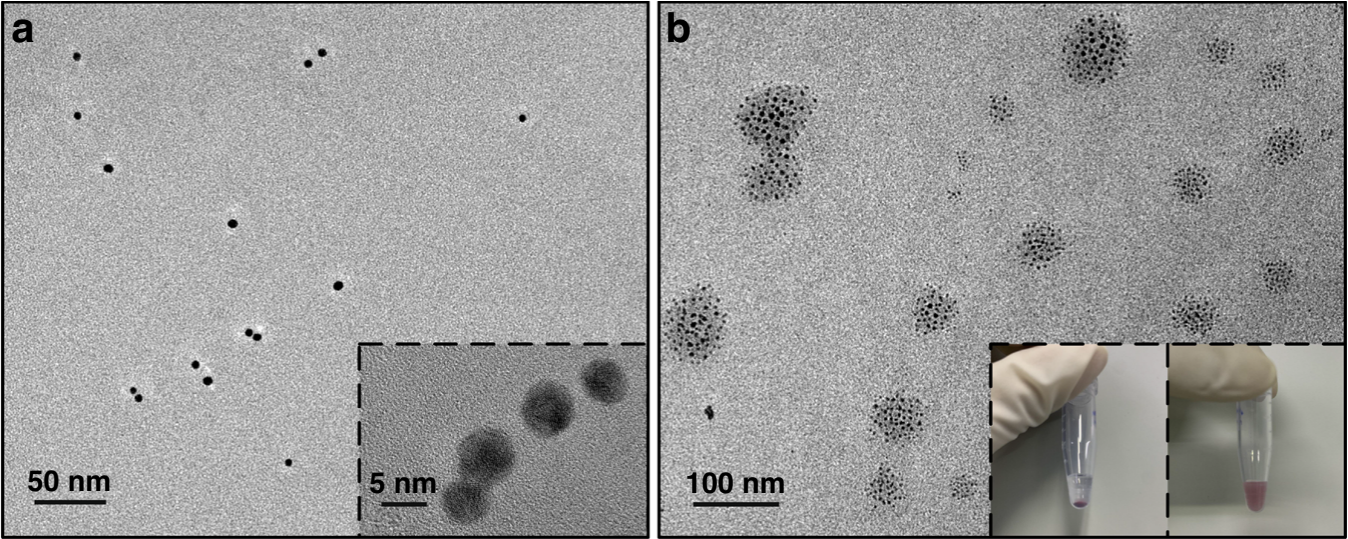
TEM images of gold nanoparticles and PS microspheres with detection probes. **a** TEM images of synthesized gold nanoparticles (scale bar of 50 nm and 2 nm respectively), with a photo of gold nanoparticles solution inserted. **b** TEM images of synthesized microspheres with detection probes

To ensure that the surface of a single gold nanoparticle is connected to only one ssDNA, gold nanoparticles with small diameter were preferred. Reversed Turkevich method is known for better yield of small gold nanoparticles than traditional Turkevich method. To obtain gold nanoparticles with smaller diameter, tannic acid was introduced in the reversed Turkevich method^48^, which has stronger reducing power than normally used sodium citrate. Briefly, 150 mL of 2.2 mM sodium citrate and 0.1 mL of 2.5 mM tannic acid were mixed and heated to 70 °C with continuous stirring. Subsequently, 1 ml of 25 mM chloroauric acid was injected into the mixed solution above. The mixed solution turned dark immediately, and then pink orange in a few minutes, indicating the success synthesis of gold nanoparticles with small diameter. Afterwards, synthesized gold nanoparticles solution was kept heating at 70 °C for another 20 min and cooled down to room temperature under stirring. It should be noted that all the containers used in this process need to be cleaned by aqua regia first because any impurities may cause irreversible aggregation of gold nanoparticles. The images of synthesized gold nanoparticles from transmission electron microscopy (TEM) are shown in Fig. 3a (with a photo of gold nanoparticles solution inserted). From the image we can know that the diameter of our gold nanoparticles is around 5 nm.

Since both DNA and gold nanoparticles are negatively charged, the electrostatic repulsion between them will prevent the natural attachment of DNA strands to the surface of gold nanoparticles though there is a strong binding affinity of thiols to gold nanoparticles. Researchers have proved that a freeze–thaw process could effectively solve this problem^49^.

Moreover, a higher efficiency of DNA hybridization is also demonstrated by the freeze–thaw process than the traditional salt-aging strategy. Though the diameter of the synthesized gold nanoparticles is quite small, modifying only one ssDNA strand on single gold nanoparticle will still cause irreversible aggregation. Therefore, when modifying ssDNA to the surface of gold nanoparticles by freeze-thaw method, we introduced spacer DNA to avoid the aggregation of gold nanoparticles.

The purchased cTnI aptamer, ssDNA and spacer DNA oligos were resuspended in nuclease-free water into 100 μM solution. 10 μL and 70 μL of 20 mM freshly-made TCEP solution were added to ssDNA and spacer DNA solution of the same volume respectively, and left for 1 h incubation to cleave the disulfide bond of DNA. Then the DNA and cTnI aptamer were diluted into 10 μM with a folding buffer (1 mM MgCl_2_, 1 × PBS, pH 7.2) and underwent a heat treatment (5 min at 85 °C, 10 min at 25 °C and 15 min at 37 °C) to help them folding into proper secondary conformation.

To ensure there wouldn’t be more than one ssDNA strand attaching to one gold nanoparticle, the ratio of ssDNA and gold nanoparticles were prepared as 1:2 for the freeze–thaw cycle. The same amount of cTnI aptamer as the ssDNA was also added to the mixture for hybridization. The spacer DNA strands utilized could maintain the colloidal stability of gold nanoparticles. The mixed solution was left in −80 °C freezer for frozen and then at room temperature for thawing. After washing four times with 1×PBS buffer, a 50 kda filter was used to remove unbound aptamers and DNA strands.

The detection probe was attached to 10 μm streptavidin coated PS beads based on streptavidin-biotin interaction. 250 μL of 10 mg/mL streptavidin modified PS beads purchased (concentration of streptavidin is 400–500 pmol/mg) was washed with buffer solution (1 × PBS, 0.25% Tween 20) three times, and then was mixed with the detection probe solution with shaking for 1.5 h. After washing with three more times, PS beads were blocked with 1 mL of 3% BSA for 1 h. The synthesized microspheres are shown in Fig. 3b.

## Results and discussion

The detection process of our sensor is shown in Fig. 2b. Samples to be tested were firstly mixed with PS beads with detection probe for 30 min to fully capture targets. Then the mixture was injected in the microchannel for SAW separation. The fluorescence intensity of the separated PS beads was used for quantify the concentration of cTnI.

### Characterization and detection results of detection probe

**Fig. 4:**
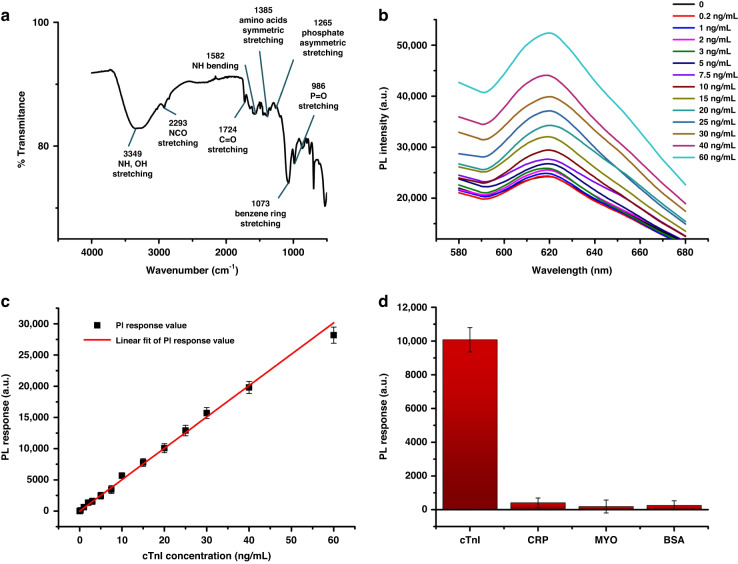
Verification of detection probes. **a** FT-IR spectra with our synthesized microspheres. **b** Fluorescence intensity curves measured by a spectrofluorometer with different cTnI concentrations. **c** Linear relationship between the PL intensity response values and concentrations of cTnI (*R*^2^ = 0.99698). **d** PL intensity response when detecting 20 ng/mL of cTnI, CRP, MYO and BSA

Fourier transform-infrared (FT-IR) spectra tests were done with our synthesized microspheres, to prove the success immobilization of detection probes. As shown in Fig. 4a, characteristic vibrations of microspheres are depicted, including the stretching vibration peak from NH and OH group at 3349 cm^−1^, the stretching vibration peak from NCO group at 2293 cm^−1^, the stretching vibration peak from C=O group at 1724 cm^−1^ and the bending vibration peak from NH group at 1582 cm^−1^. The peaks at 1385 cm^−1^, 1265 cm^−1^, 1073 cm^−1^ and 986 cm^−1^ represent the symmetric stretching vibration of the amino acids, the asymmetric stretching vibration peak of phosphate, the stretching vibration peak of styrene in polystyrene and the stretching vibration peak of P=O group, respectively^50^.

The reliability of our synthesized detection probe was verified by detecting the standard cTnI solution. Synthesized detection probe solutions were mixed with cTnI standard solutions with different known concentrations, followed by continuous shaking for 30 min. Then the mixture solution was transferred to a quartz cuvette for photoluminescence (PL) intensity testing with a spectrofluorometer. Results in Fig. 4b show that the PL intensity of the detection probes increased with the concentrations of cTnI to be measured, which verified the theoretical analysis mentioned above. The peak values of each fluorescence spectrum curve (from 0.2 to 60 ng/mL) positioned at 620 nm. The peak intensity value minus the base value (the fluorescence of the probe microspheres) was recorded as the PL intensity response. A linear relationship could be built between these response values and concentrations of cTnI within 0.2–60 ng/mL (Fig. 4c). The corresponding R^2^ was 0.99698 according to the calculation by Origin software. The detection limit of our sensor calculated by 3σ/K is 44 pg/mL in PBS buffer, where σ is the standard deviation of the blank and K is the slope of detection line. Besides, to further verify the specificity of our detection probe, proteins including C-reactive protein (CRP), myoglobin (MYO) and bovine serum albumin (BSA) were tested together with cTnI (20 ng/mL of each protein). Results in Fig. 4d show negligible PL intensity responses when detecting CRP, MYO and BSA compared to cTnI.

**Fig. 5:**
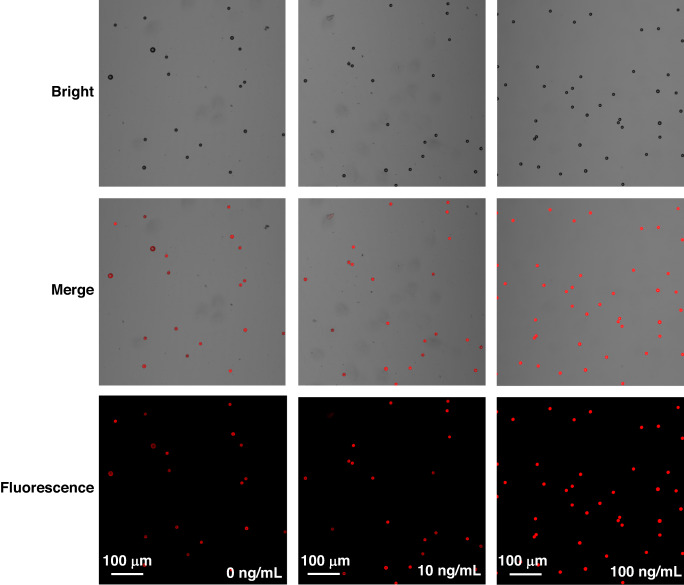
PS beads images taken by the confocal scanning microscope with 0, 10 and 100 ng/mL cTnI

In order to demonstrate more obvious change of fluorescence intensity on the surface of PS beads, 25 μL of PS beads suspension was dropped on a piece of cover glass and placed under the confocal scanning microscope. After the PS beads were fully stabilized on the cover glass, the fluorescence intensity of the microsphere was observed, as shown in Fig. 5. Images of PS beads with cTnI concentration of 0, 10 and 100 ng/mL were taken by the confocal scanning microscope, including the bright field, fluorescence field and merge field. Results showed that the fluorescence distribution on the surface of the microspheres was quite uniform. When no cTnI was added, the fluorescence intensity was quenched to a large extent. In contrast, the fluorescence intensity increased gradually as the concentration of cTnI increased to 10 and 100 ng/mL.

### SAW separation

**Fig. 6:**
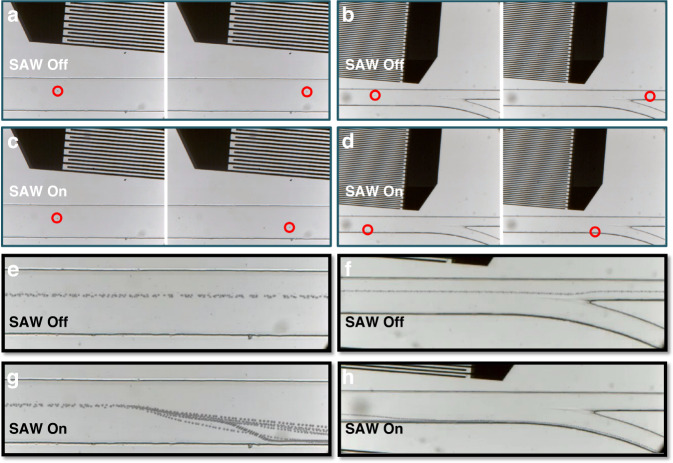
SAW separation performances. Images taken by high-speed camera of the same particle flowing through **a**, **b** the middle part of and **c**, **d** the outlet of the microchannel with and without SAW affected. Continuous particles flow trajectories in **e**, **f** the middle part of and **g**, **h** the outlet of the microfluidic channel with SAW on and off

PS beads with a diameter of 10 μm were dispersed in buffer (1 × PBS, 0.25% Tween 20) and were injected into the middle inlet of our PDMS microchannel, while PBS buffer was injected into the two side inlets. By adjusting the injection pressure of sheath on both sides, the position of PS beads in the microchannel could be controlled. An AC signal with a frequency of 48.9 MHz was applied to both ends of IDTs, and the voltage was set at 25 V to generate SAW. The images taken by high-speed camera at the entrance of SAW field and the outlet of microchannel. Figure 6 shows the movement of the microsphere suspension with and without SAW turned on.

Red circles marked positions where the same particle flowed through in the channel. It could be seen from the trajectory of the same particle that the particle passed straightly through the channel without SAW affected. When SAW was turned on, the particle would be deflected downwardly and finally flowed into outlet 2 due to the ARF perpendicular to IDTs in the downward direction. In addition, the stacked figures by Img2Go software, corresponding to the process with and without SAW, were presented also in Fig. 6e–h to obtain continuous particles flow trajectory. It showed more obviously that the introduction of SAW leaded to a good separation efficiency on 10 μm particles in a continuous period of time. The particles in microchannel were all collected by outlet 1 without effect of radiation force, while when SAW was turned on, the particles in microchannel were completely deflected and collected by outlet 2 eventually. What’ more, the SAW separation process is completely automatic as long as samples are placed in the liquid storage tanks of syringe pump.

**Fig. 7:**
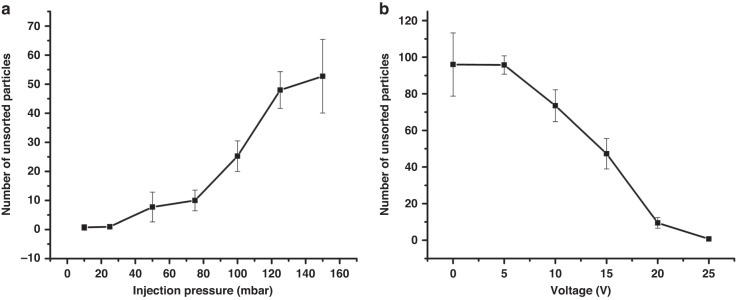
Factors affecting separation rate. **a** Relationship between separation purity and injection pressure. **b** Relationship between separation purity and voltage applied on IDTs

It is worth mentioning that a good separation purity relies on the vertical (relatively to the flow direction) shift distance of target particles. Microparticles can only be collected by outlet 2 when the vertical shift distance of target particles is large enough, which can be affected by the velocity of flow and power intensity of SAW. The faster target particles pass through SAW region, the less time ARF acts on the particles, thus, the shorter distance the particles will shift. Meanwhile, the higher voltage is applied to IDTs, the higher power intensity of SAW is. Here, we gave a demonstration of the relationship between separation purity and these two influence factors. Unsorted particles were collected and counted on a cell counting board under different injection pressure of particles and voltage applied on IDTs. Each test was repeated four times to verify the repeatability. When the voltage was fixed at 25 V, the number of unsorted particles increased with the speeding up of the flow (Fig. 7a). A good separation purity could be achieved when the injection pressure was not above 25 mbar. On the other hand, when the injection pressure was fixed at 25 mbar, particles couldn’t be completely sorted until the voltage reach 25 V (Fig. 7b). The sorting rate could be as high as 99.1% under 25 V and 25 mbar.

### Detection of cTnI in human serum

**Fig. 8:**
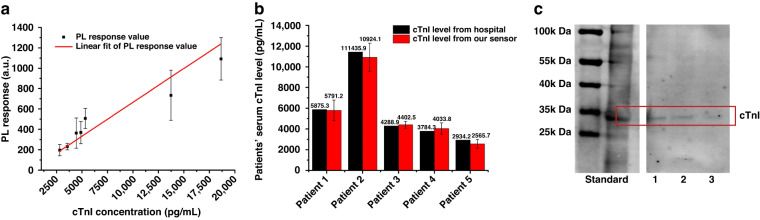
Detection of cTnI level in human serum. **a** Linear relationship between the PL intensity response and cTnI level in human serum (*R*^2^ = 0.97313). **b** Comparison results for patients’ serum tests from both hospital and our sensor. **c** Results from Western Blot experiments

Since biological molecules such as cells, vesicles and proteins which are smaller than the separation size in the body fluid samples would not be affected by SAW due to the ignorable ARF, SAW could help to exclude samples from the detection probes to effectively amplify the detection signal. The detection of cTnI concentration in human serum is similar to the process in Fig. 4, where all serum samples were obtained from The Second Affiliated Hospital of Xi’an Jiaotong University. After the reaction between detection probe and cTnI in human serum, the mixture was delivered for SAW separation. Human serum samples were 10 times diluted with PBS buffer for proper reaction ion concentration. The fluorescence of separated PS beads was tested to quantify the concentration of cTnI. Each test was repeated three times to ensure the good stability and repeatability of our biosensor at the level of experimental study. Results in Fig. 8a show a good linearity (*R*^2^ = 0.97313) between the PL intensity response and the concentration of cTnI with a slope of 0.06649. The detection limit calculated for human serum detection is 0.34 ng/mL, which is much lower than the cTnI level in AMI patients’ serum. Furthermore, five more patients’ samples were tested based on the linear relationship obtained above. Detection results from our sensor were compared with results from nephelometric analyzer in hospital in Fig. 8b. The small differences between the detection results from our sensor and hospital proved a high reliability of our sensor. Western Blot experiments was done in order to further verify the capture ability of detection probe to cTnI in human serum. However, since the level of cTnI in the patient’s serum is very low, serum samples were spiked with cTnI to obtain more obvious color reaction. As shown in Fig. 8c, from sample 1 to sample 3 were the protein captured by the probe beads from serum samples with cTnI concentrations of 200, 150, and 100 ng/mL. It could be seen that the protein captured by the detection probe and the standard one showed imprints at the same position, which verified the capture ability of our probe.

### Discussion

It is worth mentioning that performances including sensitivity, stability and repeatability are important to evaluate biosensors. However, different from other biosensors such as electrochemical method, the fluorescence signal measured by spectrofluorometer is a relative value, which varies by different spectrofluorometers and experimental settings. Thus, the sensitivity comparison of fluorescent biosensors with different experimental settings can’t represent the superiority or inferiority. In fact, very few literatures have mentioned the stability and repeatability, and most current studies are still focusing on exploring novel sensing mechanisms and rare ones have reached the application level^51–53^, which bring significant trouble to clarify the track of advancement in this field. Detection limit, instead, is more often adopted to compare the performance of biosensors in terms of sensing capacity.

By virtue of this quantitative characterization, the comparison of performances with cTnI fluorescent detection methods using different strategies to avoid background fluorescence is listed in Table 1. Actually, whether for cTnI or other low-level biomarkers, the background noise of human serum is a common challenge to fluorescent biosensors. Researchers have used different strategies to overcome it, including high dilution, filtration and washing. For the strategy of high dilution, it can significantly decrease the fluorescent noise, but also dilute the targets, limiting its ability to detect ultra-low level cTnI. While the filtration can only exclude the interference from large components, but unable to exclude fluorescence of small molecules such as proteins. Furthermore, multi-times manually washing steps do work well with the purification of targets, but also add much burden to the lab technicians. Obviously, a noise removal method with better balance between the operational complexity and detection performance is in urgent need. In this study, it has been proven that SAW separation can satisfy such kind of demand, since it enables a continuous, high-purity and labor-saving separation process. And we believe the microfluidic chips, integrating with various micro-nano manipulation techniques have great potential as core part for portable high-performance biosensing devices in the future.

**Table 1:** Comparison of different strategies to avoid background fluorescence with cTnI fluorescent detection methods

Strategy to avoid background fluorescence	Description	Detection limit	Comments
Dilution	A paper microfluidics based fluorescent immunoassay; Serum samples were diluted 50 times^54^.	19 pg/mL	Advantage: one-step, easy operation, low in chip cost; Disadvantage: didn’t mention the detection performances to test real serum samples.
	A fluorescent ELISA method; Serum samples were diluted 100 times^18^.	1 ng/mL	Advantage: in situ detection based on enzyme; Disadvantage: not sensitive enough due to dilution.
Filtration	A FRET based aptasensor; Serum samples were diluted 50 times using and filtered with centrifugal filtration^19^.	70 pg/mL	Advantage: one-step, easy operation; Disadvantage: didn’t mention the detection performances to test real serum samples.
Washing	A fluorescent ELISA method; Washing on 96-well plate to remove serum^17^.	0.081 pg/mL	Advantage: ultra-sensitive, large sample capacity; Disadvantage: complex manually operating process.
	A self-assembled microfluidic immunoassay; Washing on microfluidic chip plate to remove serum^22^.	19.37 pg/mL	Advantage: multi-biomarker detection ability, good sensitivity; Disadvantage: multiple cleaning steps necessary.
SAW separation	This work; Serum removed by SAW microfluidic chip.	44 pg/mL	An automatic process to remove background fluorescence by SAW; easy operation.

## Conclusion

This work developed a detection method for the diagnosis biomarker cTnI of AMI. Due to the extremely low level of cTnI in human serum, the traditional fluorescence detection method is limited by the strong background fluorescence interference of biological molecules in serum to obtain high sensitivity. The rapid separation to enrich cTnI based on SAW microfluidic chip proposed in this study can solve the problem properly. The aptamer detection probe synthesized by us can specifically capture cTnI in serum, and travelling surface acoustic wave is then introduced to separate the detection probe on PS beads from the body fluid sample effectively, eliminating the background noise and amplifying the detection signal. So the level of cTnI can be detected through the fluorescence intensity change. This detection method can realize rapid and accurate detection of cTnI level in human serum, and its performance has been validated by comparison with results of clinical test. What’s more, with the advantage of automatic separation process by SAW, this method has great potential for developing portable detection devices in the future.
